# Sequence Divergent RXLR Effectors Share a Structural Fold Conserved across Plant Pathogenic Oomycete Species

**DOI:** 10.1371/journal.ppat.1002400

**Published:** 2012-01-12

**Authors:** Joe Win, Ksenia V. Krasileva, Sophien Kamoun, Ken Shirasu, Brian J. Staskawicz, Mark J. Banfield

**Affiliations:** 1 The Sainsbury Laboratory, Norwich Research Park, Norwich, United Kingdom; 2 Department of Plant and Microbial Biology, University of California, Berkeley, California, United States of America; 3 Plant Science Centre, RIKEN, Tsurumi, Yokohama, Kanagawa, Japan; 4 Department of Biological Chemistry, John Innes Centre, Norwich Research Park, Norwich, United Kingdom; Duke University Medical Center, United States of America

The availability of genome sequences for some of the most devastating eukaryotic plant pathogens has led a revolution in our understanding of how these parasites cause disease, and how their hosts respond to invasion [1]–[7]. One of the most significant discoveries from the genome sequences of plant pathogenic oomycetes is the plethora of putative translocated effector proteins these organisms encode. Many effector genes display signatures of rapid evolution and tend to reside in dynamic regions of the pathogen genomes. Once inside the host, effector proteins modulate cellular processes, mainly suppressing plant immunity [8]–[12]. Effectors can also be recognized directly or indirectly by the plant immune system through the action of disease resistance (R) proteins [13], [14].

## Plant Pathogenic Oomycetes Express RXLR Effector Proteins

**Figure 1:**
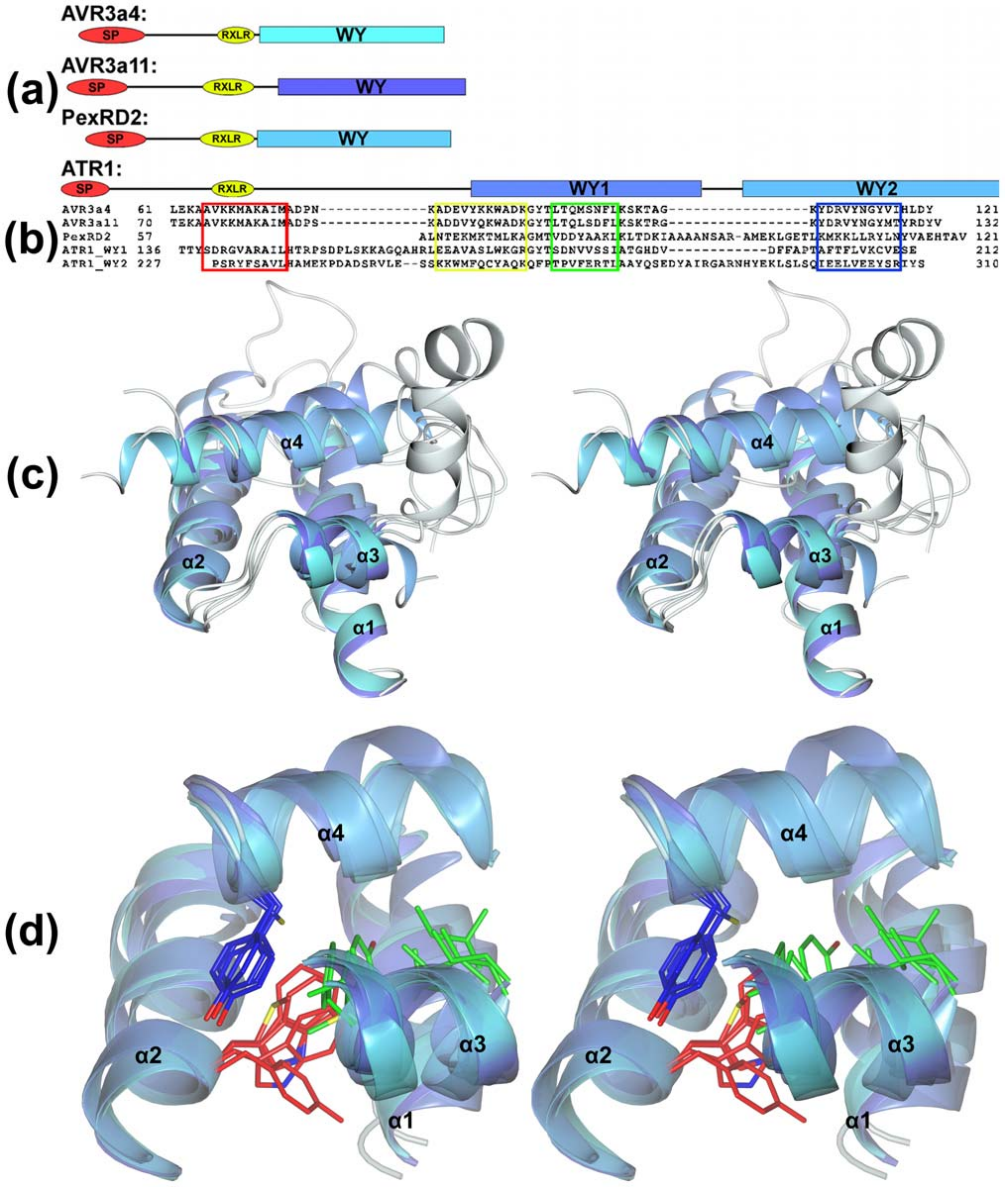
Structural conservation of the WY-domain fold in RXLR effectors from *P. infestans*, *P. capsici*, and *H. arabidopsidis*. (a) Schematic representation (to scale) of the domain architectures of AVR3a4, AVR3a11, PexRD2, and ATR1 showing the positions of the WY-domains used in the structural overlays. SP = signal peptide region, RXLR = RXLR/dEER region, WY = WY-domain regions (schematics are aligned at the end of the RXLR/dEER region). (b) Structure-based sequence alignment showing the positions of the conserved helices in each WY-domain. (c) Stereo view of an overlay comprising the WY-domains from AVR3a11, ATR1-WY1, ATR1-WY2, PexRD2, and AVR3a4 (α2–α4 span the WY-domain, α1 is the N-terminal helix present in all but PexRD2). The helices of the structures are colored in grades of blue through to cyan. Connecting regions are in gray (all structures). (d) Stereo view (orientation as in (c)) showing the positions of important residues within the hydrophobic core of the WY-domain (red = the W position, blue = the Y position, in green are two positions contributed from α3). Only the conserved helices are shown in cartoon representation (connecting regions removed for clarity).

One expanded family of effector proteins is defined by the sequence RXLR (Arg-X-Leu-Arg, where X is any amino acid), which in some cases is followed by an acidic-rich dEER motif (Asp-Glu-Glu-Arg) (Figure 1). The RXLR motif was originally identified by comparing sequences of effectors from *Hyaloperonospora arabidopsidis*, *Phytophthora infestans*, and *Phytophthora sojae* [15]. It has since been shown that the RXLR motif is important for translocation of oomycete effectors into plant cells [16], [17]. It is widely accepted that RXLR effectors are modular proteins comprising an N-terminal secretion signal, followed by the RXLR region, and a C-terminal “effector” domain that encodes the biochemical activity of the protein when expressed directly in plant cells [18], [19]. A large family of *Phytophthora* RXLR effectors contain conserved sequence motifs (W, Y, and L) in their C-terminal domains that often form tandem repeats [2], [20].

## Structural Biology Uncovers an Effector Fold Conserved across Oomycete Species

Our laboratories have employed structural biology to investigate the molecular basis of RXLR effector function. A total of four structures have recently been published, those of AVR3a4 and AVR3a11 (paralogues from *Phytophthora capsici*), PexRD2 (from *P. infestans*), and ATR1 (from *H. arabidopsidis*) [21]–[23]. Each publication focused on a different aspect of structure/function analysis including phospholipid binding, protein folding, and effector recognition by the host.

The studies of Boutemy et al. and Chou et al. independently described the structural homology of AVR3a11 and a domain of ATR1, respectively, to the cyanobacterial four-helix bundle protein KaiA [24]. This strongly implied they would also be structurally related to each other. This is unexpected, as these *Phytophthora* and *H. arabidopsidis* effectors do not share any significant sequence similarity: the conservation was only apparent after the structures were determined and compared. Further, the structural conservation across different oomycete species was particularly intriguing, as studies with the *Phytophthora* proteins AVR3a11 and PexRD2 [21] had suggested a three-helix bundle fold could be the basic structural unit adopted by the repeating W-Y motifs found in >520 *Phytophthora* RXLR effectors (44% of annotated RXLR effectors in *P. infestans*, *Phytophthora ramorum*, and *P. sojae*). Using Hidden Markov Model (HMM)-based sequence searches, these motifs had also been detected in *H. arabidopsidis* RXLR effector proteins, with 35 out of 134 (26%) containing this fold (HMM score>0), including ATR1 with a low confidence score [21]. Boutemy et al. named this conserved structural unit the “WY-domain” and it comprises three α-helices connected by variable loop regions. The minimal three-helix WY-domain is found in PexRD2, but Avr3a4, Avr3a11, and ATR1 all have an N-terminal helix as an extension to this unit that forms a four-helix bundle. Further analysis of the ATR1 structure revealed that not only residues 139–210 (the domain originally identified as having a KaiA-like fold), but also 226–308 comprised a WY-domain four-helix bundle (ATR1 also has a fifth helix that creates a five-helix repeat) [22]. This tandem repeat could not be detected from amino acid sequence comparison, and was only discovered after the ATR1 structure was determined [22]. The structure of ATR1 shows how tandem WY-domains encoded by very divergent amino acid sequences are linked in three-dimensional space. This is significant, as it provides insight into how WY-domains may be arranged in other WY motif repeat RXLR effectors.

## The Conserved Fold Is Based on a “Flexible” Hydrophobic Core

The availability of these four oomycete RXLR effector domain structures, from three different pathogens, allows us to present a detailed analysis of the WY-domain fold. Structural overlays of the conserved WY-domains from each of the effectors are shown in Figure 1, and the root mean square deviations derived from the overlays are given in Table 1 (obtained using Secondary Structure Matching (SSM) algorithms [25]). What are the features of this fold that allow structural conservation with little, if any, identifiable pair-wise sequence identity? In the HMM models, the conserved motif is largely defined by residues such as the W and Y (for Trp and Tyr) that, in each structure, are buried in the hydrophobic core of the helical bundle (other hydrophobic residues that contribute to the core are also prevalent in the HMM). Critically, the identity of these residues can change, without affecting the fold, as long as their hydrophobic potential is maintained. For example, in the WY-domain structures available, the W and Y positions are Trp-Tyr (AVR3a4 and AVR3a11), Met-Tyr (PexRD2), and Trp-Cys/Tyr-Tyr (for the two WY-domains of ATR1). Further, there is evidence from the existing structures that solvent remains excluded from the hydrophobic core when mutations from bulkier to smaller side chains occurs through complementary mutations at other positions that fill the available space. The ability of this structural fold to accommodate the rapid evolution of protein sequence explains why the WY-domain was not identifiable in pair-wise sequence comparisons.

**Table 1:** Root mean square deviations (based on C_α_ atoms) for overlays of the published RXLR effector structures (only the A-chains of ATR1 and PexRD2 were considered).

Effector	ATR1-WY1	ATR1-WY2	AVR3a4	PexRD2
**AVR3a11**	1.72 Å	1.86 Å	0.85 Å	0.73 Å
**ATR1-WY1**		2.12 Å	1.75 Å	1.30 Å
**ATR1-WY2**			1.83 Å	0.94 Å
**AVR3a4**				0.93 Å

Residues used in the overlays are those within the conserved helices of each structure as revealed by pair-wise comparisons.

## The WY-Domain Fold Is Restricted to the Peronosporales

**Figure 2:**
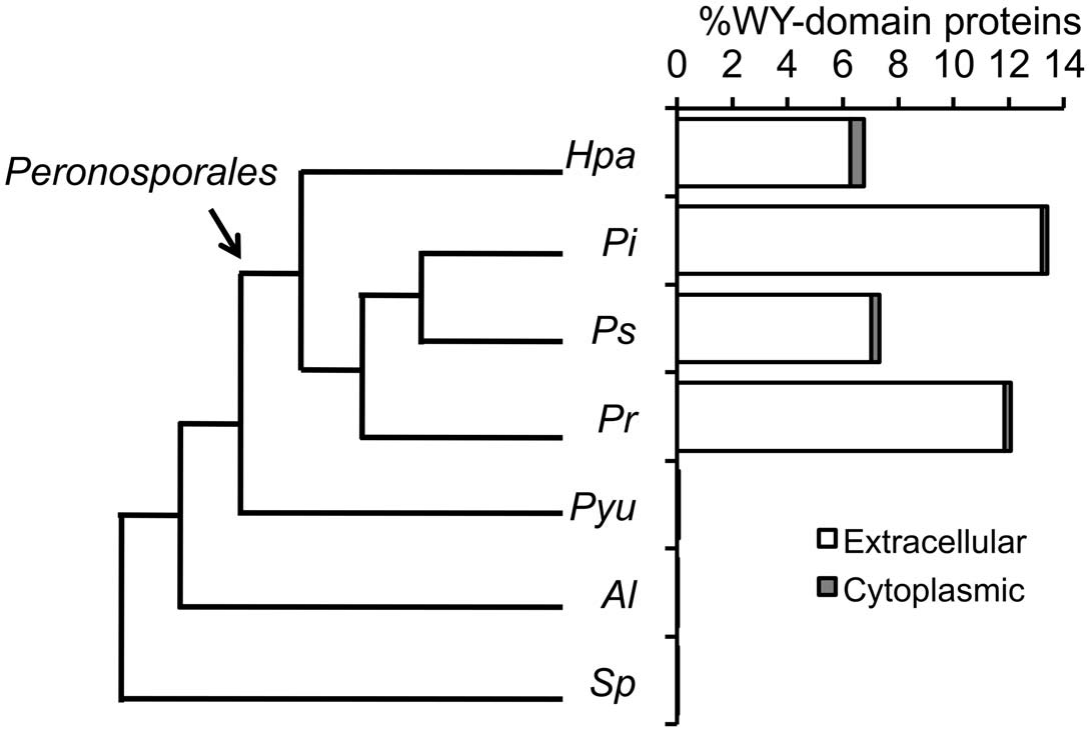
Phylogenetic relationship and presence of the WY-domain HMM signature in sequenced oomycete genomes. Schematic representation of the phylogenetic relationship among seven oomycetes for which genome sequences are available and the distribution of WY-domain in their extracellular and cytoplasmic proteomes (as a percentage of the total). *Hpa* = *H. arabidopsidis*; *Pi* = *P. infestans*; *Ps* = *P. sojae*; *Pr* = *P. ramorum*; *Pyu = P. ultimum*; *Al* = *A. labachii*; *Sp* = *S. parasitica*.

We believe that the conservation of this “flexible” hydrophobic core fold indicates that a large family of plant pathogenic oomycete effectors may have been derived from a common ancestor. Intriguingly, this effector family has rapidly diverged to gain new and/or adapt existing virulence functions and/or evade detection by plant immune systems. New or modified effector functions may be derived from surface point mutations or indels in the connecting regions between helices (including domain duplication). To test the argument for a common ancestor, we extended previous analyses and searched the proteomes of various organisms using the HMM for the WY-domain, as described in [21]. Firstly, we searched the proteomes of 47 different eukaryotes [26]. These searches included diverse species, from fungi through plants and animals. We found no evidence for the presence of the WY-domain signature beyond the level of our previously described false-positive rate [21]. We then narrowed our search and screened the available proteomes of phylogenetically diverse oomycetes: *Saprolegnia parasitica* [27], *Albugo labachii* [28], and *Pythium ultimum* [29], adding to the *Phytophthora* and *Hyaloperonospora* proteomes searched previously [21] (Figure 2). These searches show that, with the data available, the WY-domain is limited to a single clade within the oomycetes, the Peronosporales, that are exclusively plant pathogens [30]. This suggests that the WY-domain may be an innovation within the Peronosporales. The WY-domain is also correlated with the emergence of the RXLR motif in the Peronosporales, and is linked with the evolution of haustoria as a possible interface for effector delivery in this lineage [19], [29]. Of the seven oomycetes whose genome sequences are available, all of the Peronosporales (four species) have RXLRs and WY-domains (Figure 2); the non-Peronosporales (three species) encode essentially no RXLR or WY-domain proteins, and those few identified may be false positives given that they are not enriched in the secretome (as in the Peronosporales). It is also notable that oomycetes with expansions of their RXLR effector repertoire (*P. infestans*>*P. sojae*>*P. ramorum*>*H. arabidopsidis*) also encode a significantly higher percentage of WY-domains in their secretomes (*P. infestans*>*P. ramorum*>*P. sojae*>*H. arabidopsidis*, Figure 2). Further, as WY-domains are found in both *Phytophthora* (hemibiotrophs) and *Hyaloperonospora* (obligate biotrophs), but not *P. ultimum*, it appears that the WY-domain emerged with biotrophy in this lineage along with the evolution of haustoria and RXLR effectors [19], [31]. Whilst arguing in favour of a common ancestor of the WY-domain within the Peronosporales, we acknowledge that alternative interpretations (including convergent evolution to a fold adapted for stability in the plant cell and/or well-suited to a particular function, such as secretion and/or translocation) remain possible.

Some of the most notorious and agriculturally important pathogenic oomycetes contain RXLR:WY-domain effectors, suggesting that this structure has been critical for the success of these pathogens. This raises questions such as, why has this fold been preserved and what can it tell us about the function of these proteins? How can we use this knowledge to design novel disease management strategies? Future studies will help define the roles of the WY-domain fold in the virulence mechanisms of these pathogens, in particular how it engages with plant cell targets, and will help to unravel the extent of structural diversity in RXLR effectors. Our initial studies have laid the foundation for new, exciting discoveries addressing the function of oomycete effectors.
